# Evaluating age-related change in lip somatosensation using somatosensory evoked magnetic fields

**DOI:** 10.1371/journal.pone.0179323

**Published:** 2017-06-15

**Authors:** Hiroki Hihara, Hiroyasu Kanetaka, Akitake Kanno, Satoko Koeda, Nobukazu Nakasato, Ryuta Kawashima, Keiichi Sasaki

**Affiliations:** 1 Division of Advanced Prosthetic Dentistry, Tohoku University Graduate School of Dentistry, Sendai, Miyagi, Japan; 2 Liaison Center for Innovative Dentistry, Tohoku University Graduate School of Dentistry, Sendai, Miyagi, Japan; 3 Institute of Development, Aging and Cancer, Tohoku University, Sendai, Miyagi, Japan; 4 Graduate School of Tokyo Medical and Dental University, Oral and Maxillofacial Surgery, Tokyo, Japan; 5 Department of Epileptology, Tohoku University School of Medicine, Sendai, Miyagi, Japan; Boston Children's Hospital / Harvard Medical School, UNITED STATES

## Abstract

Somatosensory evoked fields (SEFs) to electrical stimulation on the right and left sides of the lower lip were measured using magnetoencephalography and compared in the bilateral hemispheres of 31 healthy normal young and 29 healthy normal elderly subjects to evaluate age-related change in lip somatosensation. The initial peak of the response around 13 ms, designated as N13m, and the second peak of the response, designated as P21m, were investigated. The N13m response, which was detected in 22 of 62 hemispheres in young adults and 37 of 58 hemispheres in elderly adults, showed significantly prolonged latency and increased equivalent current dipole (ECD) moment in the elderly adults. The P21m response, which was detected in 56 of 62 hemispheres in young adults and in 52 of 58 hemispheres in elderly adults, showed longer peak latency in the elderly adults. No significant difference was found in the ECD moment for P21m, which suggests that aging affected the SEFs of the lip somatosensation, but the effects of aging on N13m and P21m differed. Prolonged latency and increased ECD moment of N13m might result from decreased peripheral conduction and increased cortical excitation system associated with aging. Therefore, the initial response component might be an objective parameter for investigating change in lip function with age.

## Introduction

Lip somatosensation is extremely important for daily life because of the involvement in stomatognathic functions, such as swallowing, speaking, and feeding, as reflected in the large lip representation in the model of somatotopic functional organization of the primary somatosensory cortex (S1) called the homunculus [1]. The lower lip is innervated by the inferior alveolar nerve. Inferior alveolar nerve injuries sometimes occur after dental treatment and patients may lose sensation in the affected areas. Such loss of lip somatosensation can cause dysfunction of speech, mastication, and swallowing [2]. Therefore, lip somatosensation and function are thought to be interrelated to a considerable degree. In addition, decreased lip somatosensation with aging may cause dysphagia and speech disorder [3, 4]. These adverse events are important issues clinically. In particular, evaluation of age-related change in lip somatosensation is important to predict functional changes of the oral region.

Lip somatosensation is generally evaluated using psychophysical methods such as the two point discriminator and von Frey filaments [5, 6]. Various studies of age-related changes in lip somatosensation have revealed that aging decreases sensory function [3, 7, 8]. However, these methods require the cooperation of the subject and depend on subjective interpretation. Furthermore, no diagnostic index of functional disorder of the oral region during aging has been established.

Functional brain imaging techniques, especially magnetoencephalography (MEG), a non-invasive brain imaging technique, can detect the weak magnetic fields caused by neural activity. Such magnetic fields are not distorted by the scalp structure. MEG has high temporal resolution similar to electroencephalography, but MEG has higher spatial resolution. MEG detection of somatosensory evoked fields (SEFs) caused by stimulation of the median nerve is well established in clinical use.

Lip somatosensation has been investigated using SEFs for lips by several research groups, but only in young subjects [9–19]. No study has evaluated age-related changes in lip somatosensation using SEFs or other functional brain imaging techniques in elderly subjects. Age-related changes have been studied using various functional brain imaging techniques with median nerve stimulation [20–26]. These studies identified prolonged latency and increased amplitude in elderly subjects. In particular, MEG detection of the SEFs for median nerve stimulation showed prolonged latency and increased amplitude were characteristic of the initial response in elderly subjects [27–29].

The initial N20m response of the median nerve is considered to be a glutamate-dependent excitatory component [30–32], and the first component of the lip response is considered to be analogous based on the orientation of the dipole [13]. The second P35m response of the median nerve is absent in infants with immature inhibitory gamma-aminobutyric acid (GABA)-dependent component [30, 33, 34], and in patients with Angelman's syndrome, a disorder of GABAergic-related genetic involvement [35]. Consequently, the P35m is considered to be a post-excitatory inhibitory GABA-dependent component [30–32]. Similarly, the second component of the lip response is considered to be analogous based on the orientation of the dipole [11, 13, 17]. Therefore, we hypothesized that the first component of the lip response is glutamate-dependent excitatory, and the second component is post-excitatory inhibitory GABA-dependent.

Investigation of age-related change using SEFs for median nerve stimulation showed prolonged latency and increased amplitude of the N20m [27–29], and either a large equivalent current dipole (ECD) moment [29] or no effect [28] on the P35m in elderly people. Prolonged latency in elderly people can be explained by decreased peripheral conduction velocity in the spinal cord [20, 36, 37]. In addition, reduced GABAergic inhibition and enhanced glutamatergic excitation have been reported in elderly people [38–42]. Furthermore, the first component of the SEF showed larger response in patients with mild cognitive impairment than in normal elderly subjects, which can represent a diagnosis index for functional change [43]. Therefore, we hypothesized that the first component of the SEF response to lip stimulation would show prolonged latency and increased moment and the second component would show prolonged latency and decreased moment in elderly subjects.

To confirm this hypothesis, the present study investigated the location and age-related change in the first and second components of the SEF responses to lower lip stimulation using MEG.

## Subjects and methods

This article complies with the Strengthening the Reporting of Observational Studies in Epidemiology (STROBE) guidelines for reporting findings.

This study examined 62 hemispheres of 31 right-handed healthy normal young adults (YA) (22 males and 9 females; age 20–27 years, mean 22.5 years) and 58 hemispheres of 29 right-handed healthy normal elderly adults (EA) (17 males and 12 females; age 63–76 years, mean 71.0 years). No subject had a history of neurological disease. Written informed consent was obtained from all participants. This study protocol was approved by the ethical committee of the Tohoku University Graduate School of Dentistry (protocol number: 23–20) in accordance with the Declaration of Helsinki.

**Fig 1:**
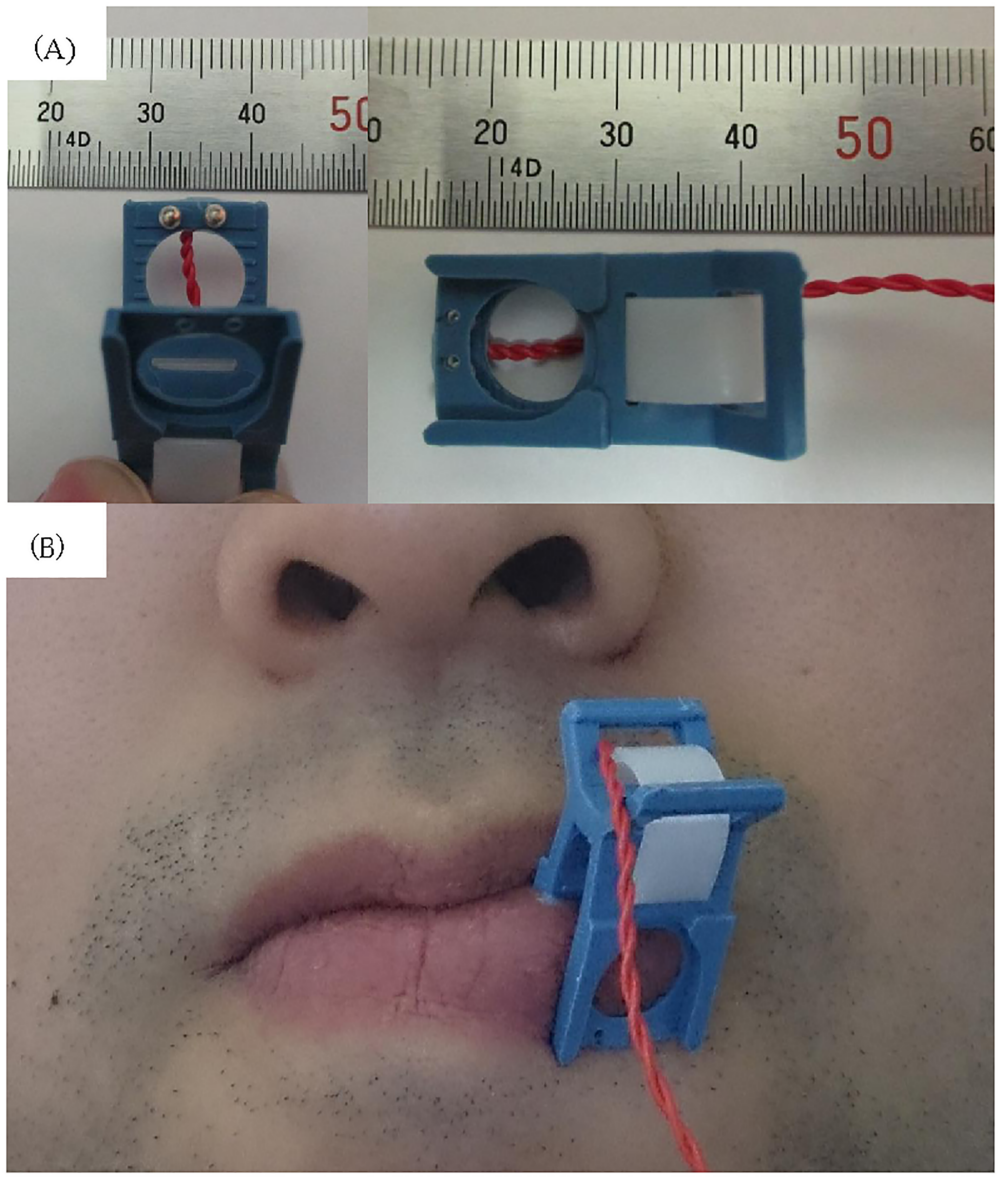
Silver ball electrodes. (A) Handmade stimulator (silver ball electrode embedded in the handmade clip) and (B) application of the electrode to the left side of the lip.

Electrical stimulation was administered to the right and left sides of the lower lip of each subjectsubjects using a handmade clip with a silver-ball electrode (Unique Medical Co., Ltd., Tokyo, Japan) attached to the surface of the mucosa facing the canines (Fig 1). The electrical stimuli consisted of constant current biphasic pulses with duration of 0.3 ms delivered at 0.7 Hz. The sensory threshold was determined by the psychophysical method. The test was conducted several times with ascending and descending series of stimulus, and the mean of the detected thresholds was considered as the sensory threshold. The stimulus intensity was below the pain threshold. No participant reported feeling any pain.

The SEF signals were measured using a whole-head 200-channel MEG system (PQA160C; Ricoh Co., Ltd., Tokyo, Japan) in a magnetically shielded room. The subjects lay supine, with the head location determined by the positions of five fiduciary markers consisting of induction coils placed at known locations on the scalp. The head shape and coil positions were established using a three-dimensional digitizer (FastSCAN Cobra; Polhemus, Inc., Colchester, VT) based on three-dimensional magnetic resonance (MR) images obtained for all subjects using a 3T MR system (Achieva; Philips Healthcare, Best, the Netherlands). The MEG signals were recorded from 50 ms before to 300 ms after the trigger point and were filtered from 0.5 to 1000 Hz, and digitized at 2000 Hz. Data for about 400 stimuli were averaged.

A previous study designated the first component of the response as N15m, observed as a contralateral initial peak with latency of around 15 ms and anterior dipole orientation [12], but used a different MEG system (Neuromag Vector View; Elekta AB, Stockholm, Sweden) to that in our study. The filter in that system delayed the latency by about 2 ms. Therefore, in the present study, the first component of the response was designated as N13m. The second component of the response was designated as P21m, a contralateral second peak with latency of around 20–25 ms with posterior dipole orientation [17]. Measurements of these N13m and P21m peaks were used for further analysis.

The SEFs were modeled as ECDs. The ECDs were used to estimate the location and moment of the source, and were superimposed on the MR images. The ECD was calculated using analysis software (Meglaboratory; Ricoh Co., Ltd.) based on Sarvas law [44] which is a method of estimating the sources of magnetic signals in a spherical conductor. All ECDs were located on the central sulcus. Goodness-of-fit greater than 70% was used for additional analyses.

The latency and single ECD moments for N13m and P21m were compared between the YA group and EA group. Additionally, the stimulus intensity and interpeak latency were compared by unpaired Student's t-tests, and the detection rate was compared by chi-square test. For multiple comparisons, the false discovery rate control test was applied to the *p*-values obtained from the Student's t-tests and chi-square tests [45, 46]. Spearman’s correlation coefficients were used to analyze the relationships between the ECD moment and the stimulus intensity, peak latency, and peak intensity. The locations of N13m and P21m were compared within the YA and EA groups. The Wilcoxon signed-rank test was used for statistical comparison. These data are expressed as the mean and standard deviation. Differences were considered significant for *p*<0.05.

Early components are known to be difficult to detect because of artifacts [9–12, 14–18]. Reportedly, no inter-hemispheric difference exists in the lip region [17]. The present study found no significant inter-hemispheric differences in ECD moment and peak latencies for N13m and P21m in the YA and EA groups.

## Results

The N13m response to lower lip stimulation was detected in 22 hemispheres of 17 YA subjects and 37 hemispheres of 25 EA subjects. The P21m response was detected in 56 hemispheres of 29 YA subjects and 52 hemispheres of 26 EA subjects. Clear responses were detected only in the contralateral hemisphere to the stimulus side; ECDs were estimated in the central sulcus.

The detection rates of N13m responses were significantly higher for EA subjects than for YA subjects (YA at 35.4% vs. EA at 63.8%; *t* = 8.7, *p* = 0.0032, chi-square test). However, the detection rates of P21m responses showed no significant difference (YA at 90.3% vs. EA at 89.7%; *t* = 0.014, *p* = 0.90).

Stimulus intensity showed no significant difference between YA and EA subjects (YA at 4.89±2.86 mA vs. EA at 4.60± 2.30 mA; *t* = 0.57, *p* = 0.56) (Table 1). Additionally, sensory threshold showed no significant difference between YA and EA subjects (YA at 0.68±0.24 mA vs. EA at 0.8± 0.52 mA; *t* = 1.6, *p* = 0.11).

**Table 1:** Mean latencies, ECD moments, stimulus intensity, and interpeak latency in each subject group.

	YA group	EA group	p Value
**N13m**			
** Latency (ms)**	12.5±1.05	15.1±1.07	<0.0001*
** ECD moment (nAm)**	2.19±0.81	6.06±2.68	<0.0001*
** Detection rate (%)**	35.4	63.8	0.0032*
**P21m**			
** Latency (ms)**	20.5±2.03	21.7±1.69	0.0016*
** ECD moment (nAm)**	13.3±9.06	11.4±5.73	0.19
** Detection rate (%)**	90.3	89.7	0.90
**Stimulus intensity (mA)**	4.89±2.86	4.60±2.30	0.56
**Interpeak latency (ms)**	8.28±1.32	7.08±1.42	0.0030*

Significant differences between subject groups.

*p<0.05.

**Fig 2:**
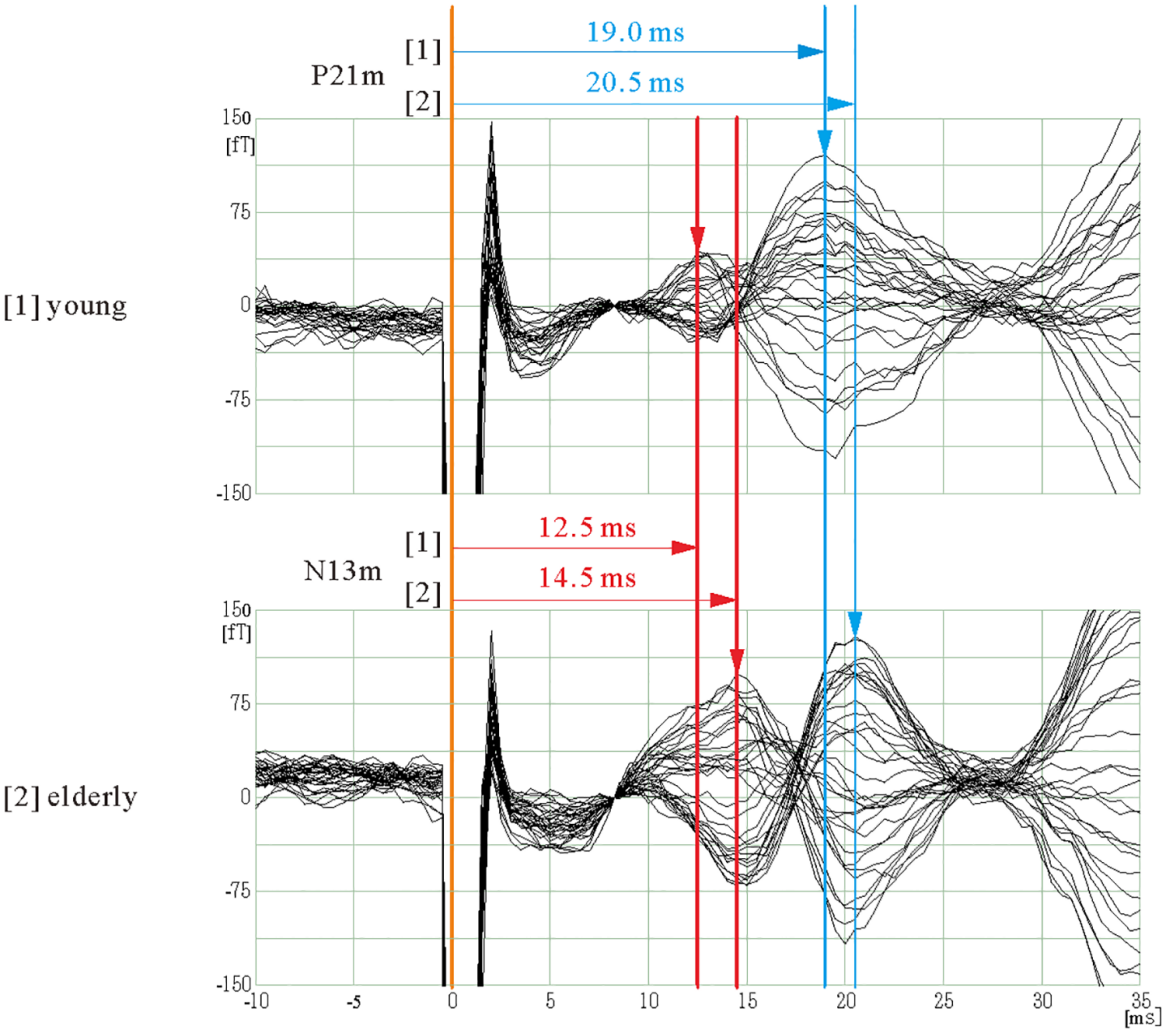
Waveforms and latencies for N13m and P21m after right side stimulation. [1] YA subject (25 years old, male), [2] EA subject (71 years old, male). Each waveform shows 10 ms before to 35 ms after stimulus onset. Red and blue arrows indicate peak latencies for N13m and P21m, respectively.

Fig 2 presents examples of the waveforms and latencies for N13m and P21m in each group. Significant age effects were found in the latencies for both N13m and P21m. The latency of N13m was significantly longer for EA subjects than for YA subjects (YA at 12.5±1.05 ms vs. EA at 15.1±1.07 ms; *t* = 9.0, *p*<0.0001). In addition, the latency of P21m was significantly longer for EA subjects than for YA subjects (YA at 20.5±2.03 ms vs. EA at 21.7±1.69 ms; *t* = 3.2, *p* = 0.0016). Interpeak latency was significantly longer for YA subjects than for EA subjects (YA at 8.28± 1.32 ms vs. EA at 7.08± 1.42 ms; *t* = 3.1, *p* = 0.0030)

**Fig 3:**
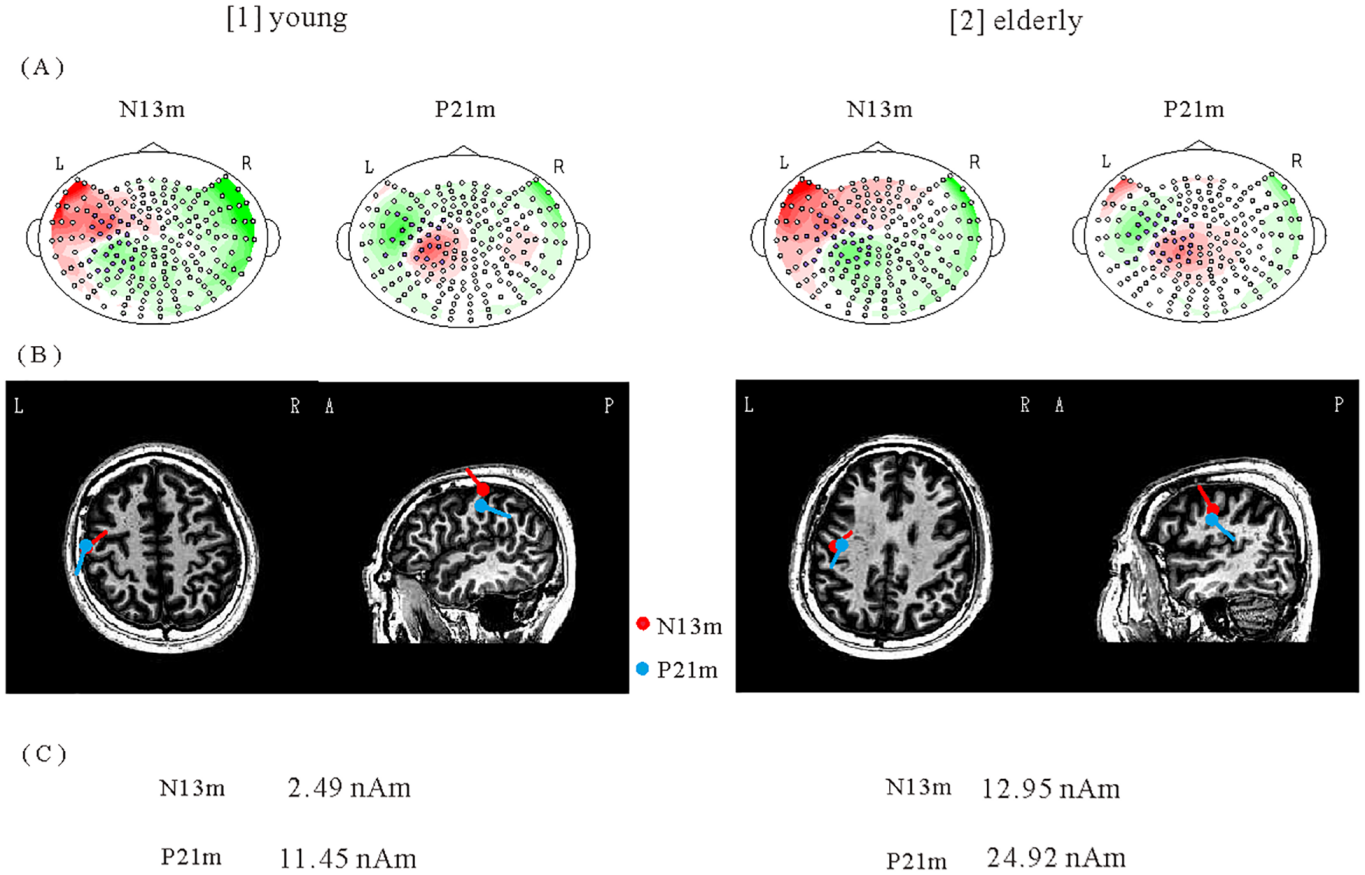
ECD locations and moments of N13m and P21m. [1] YA subject (25 years old, male), [2] EA subject (71 years old, male). (A) Isofield maps. (B) ECD locations. (C) ECD moment of N13m shows a larger moment in the EA subject than in the YA subject.

Fig 3 shows isofield maps and ECD locations for N13m and P21m. ECD moments for N13m indicated a significant age effect. The ECD moment of N13m for EA subjects was significantly larger than for YA subjects (YA at 2.19±0.81 nAm vs. EA at 6.06±2.68 nAm; *t* = 8.2, *p*<0.0001). However, the ECD moment of P21m showed no significant difference (YA at 13.3±9.06 nAm vs. EA at 11.4±5.73 nAm; *t* = 1.3, *p* = 0.19). The correlation between ECD moment and stimulus of P21m for YA subjects was significant (P21m for YA: *r* = 0.37, *p* = 0.0054). No other significant correlation was found between the ECD moment and the stimulus intensity (N13m for YA: *r* = 0.17, *p* = 0.44; N13m for EA: *r* = 0.31, *p* = 0.069; P21m for EA: *r* = 0.037, *p* = 0.79). Furthermore, no significant correlation was found between latency and stimulus intensity (N13m for YA: *r* = 0.12, *p* = 0.59; N13m for EA: *r* = 0.18, *p* = 0.30; P21m for YA: *r* = 0.13, *p* = 0.32; P21m for EA: *r* = -0.0054, *p* = 0.70).

Locations of N13m and P21m were compared within each group. The locations of N13m were significantly different from those of P21m in the Z axis and θ for YA subjects (Z: *p* = 0.0074, θ: *p*<0.0001) and θ for EA subjects (θ: *p*<0.0001) (Table 2).

**Table 2:** Location (XYZ) and orientation (deg) for N13m and P21m in each subject group.

	YA group				EA group			
	Left		Right		Left		Right	
	N13m	P21m	N13m	P21m	N13m	P21m	N13m	P21m
X(mm)	48.9±6.74	44.1±7.19	-47.6±7.74	-42.4±8.49	48.7±7.31	47.8±6.29	-47.8±9.47	-43.2±6.77
Y(mm)	7.96±10.4	12.1±11.0	1.70±10.4	10.5±12.3	10.1±11.2	11.4±12.2	9.30±6.73	9.45±6.33
Z(mm)	77.3±4.50*	66.9±7.13	72.1±10.6*	68.8±9.58	62.8±7.21	60.2±10.0	64.7±12.0	62.5±10.3
θ(°)	60.1±18.9*	111±23.9	55.3±29.7*	101±16.6	64.3±18.3*	110±16.4	56.4±18.5*	119±13.0

Significant differences between N13m and P21m on the same side in each subject group

*p<0. 01

## Discussion

The present study tested our hypothesis that the first component of the SEF response to lip stimulation would show prolonged latency and increased moment and the second component would show prolonged latency and decreased moment in elderly subjects. The present MEG study of age-related change in lip somatosensation showed markedly prolonged latency and increased ECD moment for N13m and prolonged latency for P21m in the EA subjects compared to the YA subjects. However there was no significant difference in ECD moment for P21m. These findings agreed with our hypothesis for the latency and moment of the first component (N13m), and the latency for the second component (P21m). However, the finding for the ECD moment for P21m was not consistent with the hypothesis.

Previous studies were based on analysis of the P21m or later components [9–12, 14–18]. In the present study, the P21m was observed in almost all subjects. However, the N13m was more difficult to detect because of its low amplitude and the effects of stimulus artifacts. Specifically, the N13m was detectable in 37 of 58 hemispheres of EA subjects, but in only 22 of 62 hemispheres of YA subjects. Detection of the N13m was significantly higher in EA subjects than in YA subjects. The amplitude of N13m was lower in YA subjects than in EA subjects. Additionally, the latencies of N13m were shorter in YA subjects than in EA subjects. Therefore, the N13m of YA subjects was more affected by artifacts. These reasons explain the fewer detections of N13m in YA subjects.

N13m and P21m were only detected in the contralateral hemisphere to the stimulus side, as reported previously [11, 13, 17]. However, the ECDs of the first and second components were estimated bilaterally [4], possibly due to the use of tactile stimuli in contrast to electrical stimuli used in other studies including the present study. Therefore, the stimulus methods might affect such interhemispheric differences.

Prolonged latency and increased ECD moment of N13m, and prolonged latency of P21m were observed in the EA subjects, as we hypothesized. The absence of significant correlation between ECD moment and stimulus intensity for N13m, and between the latency and stimulus intensity for N13m and P21m showed that the changes are related to aging and not to the stimulus intensity. The higher detection rate of N13m in EA subjects might be related to this increased response.

The N13m response is considered to be an excitatory glutamate-dependent component like the N20m response to median nerve stimulation. The N20m pathway is known to input directly to the primary somatosensory cortex via the thalamus without the involvement of synapses [47–50]. Therefore, the N13m pathway probably also inputs directly to the primary somatosensory cortex via the thalamus without synapses, so directly reflects the decreased peripheral conduction and enhancement of glutamatergic excitation with aging. Accordingly, N13m may provide a parameter for measuring change with aging.

Median nerve studies have suggested that the N20m and P35m responses are generated independently [51, 52]. Other studies also suggested that the locations of N20m and P35m are different [53–55]. Consequently, the pathways of N20m and P35m from the peripheral to primary somatosensory cortices are likely to be different. The P35m response is known to be generated by inhibition in the deeper cortical layer [31, 32, 56, 57]. Therefore, P21m input to the primary somatosensory cortex occurs via synapses. Long latency components are affected by the attention and vigilance states [58, 59]. Additionally, some sources persisted after the response of P35m [29], and the response of N20m overlaps the response of P35m [59]. Therefore, the P21m response might be affected by the N13m response, and the attention and vigilance states. Consequently, N13m might be regarded as a pure response that is not affected by the attention or vigilance state.

Interpeak latency was shorter in the EA subjects than in the YA subjects. Therefore, the prolonged latency for P21m cannot be explained by only aging change of the peripheral nerves. The greater amplitude of N13m and increased rising of the waveform result from enhancement of glutamatergic excitation. In contrast, the slightly smaller amplitude of P21m and reduced rising of the waveform result from reduction of the suppression system. The combination of these effects caused the shorter interpeak latency. This observation is thought to reflect the different signal sources of N13m and P21m, and seems to reinforce the hypothesis that the respective pathways and locations are different.

This study showed that the locations of N13m and P21m were significantly different as we hypothesized. Furthermore, the correlation between ECD moment and stimulus intensity of P21m was significant in the YA subjects. These findings support the idea that P21m is affected by the attention and vigilance states, and the activities of other cortical regions. Consequently, the location and the effects of aging differ between N13m and P21m.

In conclusion, the present study detected age-related differences in lip somatosensation using somatosensory evoked magnetic field analysis. Our findings indicate that N13m is more useful than P21m as an objective parameter of aging change in lip somatosensation, and may be useful for evaluating functional change.

## References

[1] Penfield W, Boldrey E. Somatic motor and sensory representation in the cerebral cortex of man as studied by electrical stimulation. Brain. 1937;60:389–443.

[2] Ziccardi VB, Assael LA. Mechanisms of trigeminal nerve injuries. Atlas Oral Maxillofac Surg Clin North Am. 2001;9:1–11. 11665372

[3] Wohlert AB. Tactile perception of spatial stimuli on the lip surface by young and older adults. J Speech Hear Res. 1996;39(6):1191–1198. 8959604 10.1044/jshr.3906.1191

[4] Tamura F, Fukui T, Kikutani T, Machida R, Yoshida M, Yoneyama T, et al Lip-closing function of elderly people during ingestion: comparison with young adults. Int J Orofacial Myology 2009;35:33–43. 20572436

[5] Jacobs R, Wu CH, Goossens K, Van Loven K, Van Hees J, Van Steenberghe D. Oral mucosal versus cutaneous sensory testing: a review of the literature. J Oral Rehabil. 2002;29(10):923–950. 12421324 10.1046/j.1365-2842.2002.00960.x

[6] Poort LJ, van Neck JW, van der Wal KG. Sensory testing of inferior alveolar nerve injuries: a review of methods used in prospective studies. J Oral Maxillofac Surg. 2009;67(2):292–300. doi: 10.1016/j.joms.2008.06.076 19138602 10.1016/j.joms.2008.06.076

[7] Calhoun KH, Gibson B, Hartley L, Minton J, Hokanson JA. Age-related changes in oral sensation. Laryngoscope. 1992;102(2):109–116. doi: 10.1288/00005537-199202000-00001 1738279 10.1288/00005537-199202000-00001

[8] Heft MW, Robinson ME. Age differences in orofacial sensory thresholds. J Dent Res. 2010;89(10):1102–1105. doi: 10.1177/0022034510375287 20651093 10.1177/0022034510375287 PMC3318051

[9] Disbrow EA, Hinkley LB, Roberts TPJ. Ipsilateral representation of oral structures in human anterior parietal somatosensory cortex and integration of inputs across the midline. Comp Neurol. 2003;467(4):487–495. 10.1002/cne.10935 14624483

[10] Hoshiyama M, Kakigi R, Koyama S, Kitamura Y, Shimojo M, Watanabe S. Somatosensory evoked magnetic fields following stimulation of the lip in humans. Electroencephalogr Clin Neurophysiol. 1996;100:96–104. 8617157 10.1016/0013-4694(95)00241-3

[11] Maezawa H, Matsuhashi M, Yoshida K, Mima T, Nagamine T, Fukuyama H. Evaluation of lip sensory disturbance using somatosensory evoked magnetic fields. Clin Neurophysiol. 2014;125(2):363–369. doi: 10.1016/j.clinph.2013.07.017 24035362 10.1016/j.clinph.2013.07.017

[12] Murayama S, Nakasato N, Nakahara H, Konno A, Itoh H. Neuromagnetic evidence that gingival area is adjacent to tongue area in human primary somatosensory cortex. Tohoku J Exp Med. 2005;207:191–195. 16210829 10.1620/tjem.207.191

[13] Nagamatsu K, Nakasato N, Hatanaka K, Kanno A, Iwasaki M, Yoshimoto T. Neuromagnetic localization of N15, the initial cortical response to lip stimulus. Neuroreport. 2001;12:1–5. 11201065 10.1097/00001756-200101220-00008

[14] Nakahara H, Nakasato N, Kanno A, Murayama S, Hatanaka K, Itoh H, et al Somatosensory evoked fields for gingiva, lip, and tongue. J Dent Res. 2004;83:307–311. doi: 10.1177/154405910408300407 15044504 10.1177/154405910408300407

[15] Nakamura A, Yamada T, Goto A, Kato T, Ito K, Abe Y, et al Somatosensory homunculus as drawn by MEG. Neuroimage. 1998;7:377–386. doi: 10.1006/nimg.1998.0332 9626677 10.1006/nimg.1998.0332

[16] Nevalainen P, Ramstad R, Isotalo E, Haapanen ML, Lauronen L. Trigeminal somatosensory evoked magnetic fields to tactile stimulation. Clin Neurophysiol. 2006;117:2007–2015. doi: 10.1016/j.clinph.2006.05.019 16859989 10.1016/j.clinph.2006.05.019

[17] Otsuka T, Dan H, Dan I, Sase M, Sano T, Tsuzuki D, et al Effect of local anesthesia on trigeminal somatosensory-evoked magnetic fields. J Dent Res. 2012;91:1196–1201. doi: 10.1177/0022034512462398 23018817 10.1177/0022034512462398

[18] Suzuki T, Shibukawa Y, Kumai T, Shintani M. Face area representation of primary somatosensory cortex in humans identified by whole-head magnetoencephalography. Jpn J Physiol. 2004;54(2):161–169. 15182423 10.2170/jjphysiol.54.161

[19] Tamura Y, Shibukawa Y, Shintani M, Kaneko Y, Ichinohe T. Oral structure representation in human somatosensory cortex. Neuroimage. 2008;43(1):128–135. doi: 10.1016/j.neuroimage.2008.06.040 18672075 10.1016/j.neuroimage.2008.06.040

[20] Desmedt JE, Cheron G. Somatosensory evoked potentials to finger stimulation in healthy octogenarians and in young adults: wave forms, scalp topography and transit times of parietal and frontal components. Electroencephalogr Clin Neurophysiol. 1980;50:404–425. 6160983 10.1016/0013-4694(80)90007-3

[21] Desmedt JE, Cheron G. Non-cephalic reference recording of early somatosensory potentials to finger stimulation in adult or aging normal differentiation. Electroencephalogr Clin Neurophysiol. 1981;50:404–425. 10.1016/0013-4694(81)91430-9 6172255

[22] Ferri R, Del Gracco S, Elia M, Musumeci SA, Spada R, Stefanini MC. Scalp topographic mapping of middle-latency somatosensory evoked potentials in normal aging and dementia. Neurophysiol Clin. 1996;26(5):311–319. doi: 10.1016/S0987-7053(97)85098-8 8987047 10.1016/S0987-7053(97)85098-8

[23] Hume AL, Cant BR, Shaw NA, Cowan JC. Central somatosensory conduction time from 10 to 79 years. Electroencephalogr Clin Neurophysiol. 1982;54(1):49–54. 6177517 10.1016/0013-4694(82)90230-9

[24] Kakigi R, Shibasaki H. Effects of age, gender, and stimulus side on scalp topography of somatosensory evoked potentials following median nerve stimulation. J Clin Neurophysiol. 1991;8:320–330. 1918337 10.1097/00004691-199107010-00008

[25] Kazis A, Vlaikidis N, Pappa P, Papanastasiou J, Vlahveis G, Routsonis K. Somatosensory and visual evoked potentials in human aging. Electromyogr Clin Neurophysiol. 1983;23(1–2):49–59. 6840038

[26] Touge T, Takeuchi H, Sasaki I, Deguchi K Ichihara N. Enhanced amplitude reduction of somatosensory evoked potentials by voluntary movement in elderly people. Electroencephalogr Clin Neurophysiol. 1997;104(2):108–214. 9146476 10.1016/s0168-5597(97)96136-0

[27] Hagiwara K, Ogata K, Okamoto T, Uehara T, Hironaga N, Shigeto H, et al Age-related changes across the primary and secondary somatosensory areas: An analysis of neuromagnetic oscillatory activities. Clin Neurophysiol. 2014;125:1021–1029. doi: 10.1016/j.clinph.2013.10.005 24189210 10.1016/j.clinph.2013.10.005

[28] Huttunen J, Wikstrom H, Salonen O, Ilmoniemi RJ. Human somatosensory cortical activation strengths: comparison between males and females and age-related changes. Brain Res. 1996;818(2):196–203. 10.1016/s0006-8993(98)01215-3 10082804

[29] Stephen JM, Ranken D, Best E, Adair J, Knoefel J, Kovacevic S, et al Aging changes and gender differences in response to median nerve stimulation measured using MEG. Clin Neurophysiol. 2006;117:131–143. doi: 10.1016/j.clinph.2005.09.003 16316782 10.1016/j.clinph.2005.09.003

[30] Nevalainen P, Lauronen L, Pihko E. Development of human somatosensory cortical functions—what have we learned from magnetoencephalography: a review. Front Hum Neurosci. 2014;8:158 doi: 10.3389/fnhum.2014.00158 24672468 10.3389/fnhum.2014.00158 PMC3955943

[31] Huttunen J, Hömberg V. Influence of stimulus repetition rate on cortical somatosensory potentials evoked by median nerve stimulation: implications for generation mechanisms. J Neurol Sci. 1991;105:37–43. 1795167 10.1016/0022-510x(91)90115-n

[32] Huttunen J, Pekkonen E, Kivisaari R, Autti T, Kähkönen S. Modulation of somatosensory evoked fields from SI and SII by acute GABA A-agonism and paired-pulse stimulation. Neuroimage. 2008;40(2):427–434 doi: 10.1016/j.neuroimage.2007.12.024 18234513 10.1016/j.neuroimage.2007.12.024

[33] Lauronen L, Nevalainen P, Wikström H, Parkkonen L, Okada Y, Pihko E. Immaturity of somatosensory cortical processing in human newborns. Neuroimage. 2006;33(1):195–203. doi: 10.1016/j.neuroimage.2006.06.041 16908201 10.1016/j.neuroimage.2006.06.041

[34] Pihko E, Nevalainen P, Stephen J, Okada Y, Lauronen L. Maturation of somatosensory cortical processing from birth to adulthood revealed by magnetoencephalography. Clin Neurophysiol. 2009;120(8):1552–1561. doi: 10.1016/j.clinph.2009.05.028 19560400 10.1016/j.clinph.2009.05.028

[35] Egawa K, Asahina N, Shiraishi H, Kamada K, Takeuchi F, Nakane S, et al Aberrant somatosensory-evoked responses imply GABAergic dysfunction in Angelman syndrome. Neuroimage. 2008;39:593–599. doi: 10.1016/j.neuroimage.2007.09.006 17962046 10.1016/j.neuroimage.2007.09.006

[36] Dorfman LJ, Bosley TM. Age-related changes in peripheral and central nerve conduction in man. Neurology. 1979;29(1):38–44. 570675 10.1212/wnl.29.1.38

[37] Drechsler F. Quantitative analysis of neurophysiological processes of the aging CNS. J Neurol.1978;218:197–213. 79647 10.1007/BF00313013

[38] Palmer AM, Robichaud PJ, Reiter CT. The release and uptake of excitatory amino acids in rat brain: effect of aging and oxidative stress. Neurobiol Aging. 1994;15(1):103–111. 7909140 10.1016/0197-4580(94)90150-3

[39] Post-Munson DJ, Lum-Ragan JT, Mahle CD, Gribkoff VK. Reduced bicuculline response and GABAA agonist binding in aged rat hippocampus. Neurobiol Aging. 1994;15(5):629–633. 7824055 10.1016/0197-4580(94)00057-3

[40] Saransaari P, Oja SS. Age-related changes in the uptake and release of glutamate and aspartate in the mouse brain. Mech Ageing Dev. 1995;81(2–3):61–71. 8569281 10.1016/0047-6374(95)01583-l

[41] Zou J, Wang YX, Dou FF, Lü HZ, Ma ZW, Lu PH, et al Glutamine synthetase down-regulation reduces astrocyte protection against glutamate excitotoxicity to neurons. Neurochem Int. 2010;56:577–584. doi: 10.1016/j.neuint.2009.12.021 20064572 10.1016/j.neuint.2009.12.021 PMC2831119

[42] Wenk GL, Pierce DJ, Struble RG, Price DL, Cork LC. Age-related changes in multiple neurotransmitter systems in the monkey brain. Neurobiol Aging. 1989;10(1):11–19. 2569169 10.1016/s0197-4580(89)80005-3

[43] Stephen JM, Montaño R, Donahue CH, Adair JC, Knoefel J, Qualls C, et al Somatosensory responses in normal aging, mild cognitive impairment, and Alzheimer's disease. J Neural Transm (Vienna). 2010;117(2):217–225. 20013008 10.1007/s00702-009-0343-5 PMC2833105

[44] Sarvas J. Basic mathematical and electromagnetic concepts of the biomagnetic inverse problem. Phys Med Biol. 1987;32(1):11–22. 3823129 10.1088/0031-9155/32/1/004

[45] Benjamini Y, Hochberg Y. Controlling the false discovery rate: a practical and powerful approach to multiple testing. J R Stat Soc Series B Stat Methodol. 1995;57:289e300.

[46] Glickman ME, Rao SR, Schultz MR. False discovery rate control is a recommended alternative to Bonferroni-type adjustments in health studies. J Clin Epidemiol. 2014;67(8):850–857. doi: 10.1016/j.jclinepi.2014.03.012 24831050 10.1016/j.jclinepi.2014.03.012

[47] Allison T, McCarthy G, Wood CC, Darcey TM, Spencer DD, Williamson PD. Human cortical potentials evoked by stimulation of the median nerve. I. Cytoarchitectonic areas generating short-latency activity. J Neurophysiol. 1989;62(3):694–710. 2769354 10.1152/jn.1989.62.3.694

[48] Baumgartner C, Doppelbauer A, Deecke L, Barth DS, Zeitlhofer J, Lindinger G, et al Neuromagnetic investigation of somatotopy of human hand somatosensory cortex. Exp Brain Res. 1991;87:641–648. 1783032 10.1007/BF00227089

[49] Hari R, Kaukoranta E. Neuromagnetic studies of somatosensory system: principles and examples. Prog Neurobiol. 1985;24:233–256. 3929329 10.1016/0301-0082(85)90007-3

[50] Wood CC, Cohen D, Cuffin BN, Yarita M, Allison T. Electrical sources in human somatosensory cortex: identification by combined magnetic and potential recordings. Science. 1985;227:1051–1053. 3975600 10.1126/science.3975600

[51] Goto S, Fujisawa Y, Uemura J, Yamada S, Hoshiyama M, Hirayama M. Disinhibitory shift of recovery curve of somatosensory-evoked response in elderly: A magnetoencephalographic study. Clin Neurophysiol. 2015;126(6):1228–1233. doi: 10.1016/j.clinph.2014.09.018 25449557 10.1016/j.clinph.2014.09.018

[52] Lin YY, Chen WT, Liao KK, Yeh TC, Wu ZA, Ho LT, et al Differential generators for N20m and P35m responses to median nerve stimulation. Neuroimage. 2005;25(4):1090–1099. doi: 10.1016/j.neuroimage.2004.12.047 15850727 10.1016/j.neuroimage.2004.12.047

[53] Kitamura Y, Kakigi R, Hoshiyama M, Koyama S, Nakamura A. Effects of sleep on somatosensory evoked responses in human: a magnetoencephalographic study. Brain Res Cogn Brain Res. 1996;4(4):275–279. 8957568 10.1016/s0926-6410(96)00066-3

[54] Kawamura T, Nakasato N, Seki K, Kanno A, Fujita S, Fujiwara S, et al Neuromagnetic evidence of pre- and post-central cortical sources of somatosensory evoked responses. Electromyogr Clin Neurophysiol. 1996;100:44–50. 10.1016/0168-5597(95)00217-0 8964262

[55] Papadelis C, Eickhoff SB, Zilles K, Ioannides AA. BA3b and BA1 activate in a serial fashion after median nerve stimulation: direct evidence from combining source analysis of evoked fields and cytoarchitectonic probabilistic maps. Neuroimage. 2011; 54(1):60–73. doi: 10.1016/j.neuroimage.2010.07.054 20691793 10.1016/j.neuroimage.2010.07.054 PMC8015308

[56] Wikström H, Huttunen J, Korvenoja A, Virtanen J, Salonen O, Aronen H, et al Effects of interstimulus interval on somatosensory evoked magnetic fields (SEFs): a hypothesis concerning SEF generation at the primary sensorimotor cortex. Electroencephalogr Clin Neurophysiol. 1996;100(6):479–487. 8980411 10.1016/s0921-884x(96)95688-x

[57] Restuccia D, Valeriani M, Grassi E, Gentili G, Mazza S, Tonali P, et al Contribution of GABAergic cortical circuitry in shaping somatosensory evoked scalp responses: specific changes after single-dose administration of tiagabine. Clin Neurophysiol. 2002;113(5):656–671. 11976045 10.1016/s1388-2457(02)00034-2

[58] Kakigi R, Naka D, Okusa T, Wang X, Inui K, Qiu Y, et al Sensory perception during sleep in humans: a magnetoencephalographic study. Sleep Med. 2003;4(6):493–507. 14607343 10.1016/s1389-9457(03)00169-2

[59] Huang M, Aine CJ, Supek S, Best E, Ranken D, Flynn ER. Multi-start downhill simplex method for spatio-temporal source localization in magnetoencephalography. Electroencephalogr Clin Neurophysiol. 1998;108(1):32–44. 9474060 10.1016/s0168-5597(97)00091-9

